# EEG dynamics during treatment in elderly depressive patients with organic mood disorder

**DOI:** 10.1192/j.eurpsy.2026.11418

**Published:** 2026-07-31

**Authors:** A. F. Iznak, E. V. Iznak, E. V. Damyanovich, S. E. Mirova, T. P. Safarova

**Affiliations:** 1 Laboratory of Neurophysiology; 2 Department of Geriatric Psychiatry, Mental Health Research Centre, Moscow, Russian Federation

## Abstract

**Introduction:**

The prognosis of treatment response in elderly patients suffered with organic mood disorder is rather difficult due to various cognitive and somatic complications.

**Objectives:**

The goal of the study was the analysis of spectral EEG parameters dynamics during treatment of elderly depressive patients with organic mood disorder, with the future aim to search for EEG correlates and predictors of individual treatment effectiveness.

**Methods:**

27 elderly depressive patients (18 females, 9 males, 55-83 years old, mean age 70,1 ± 7,4 years) with organic mood disorder (F06.3 by ICD-10) and mild cognitive impairments were included in the study. Pre-treatment and post-treatment clinical conditions were assessed using battery of scales (HADS, MMSE, MOCA, FAB). Resting-state EEGs were recorded before and after the treatment course with consequent analysis of absolute EEG spectral power in 8 narrow frequency sub-bands. Both clinical and EEG changes were statistically assessed using the Wilcoxon test.

**Results:**

A preliminary analysis of the data obtained showed that after one month treatment course the clinical conditions of patients improved in the form of significant decrease in depression and anxiety scores (according to HADS scale) without significant cognitive changes. Such improvement was accompanied by an increase in the spectral power of the EEG in all analyzed frequency sub-bands, which may reflect a general improvement in the blood supply to the brain. Besides, restructuring of the interhemispheric EEG asymmetry occurred. Thus, the focus of beta2 (20-30 Hz) spectral power shifted from the anterior and middle temporal leads of the right hemisphere towards the anterior temporal-central leads of the left hemisphere, which reflects greater activation of the anterior regions of the left hemisphere associated with the regulation of positive emotions, that is consistent with the reduction of symptoms of depression. In addition, a focus of delta (2-4 Hz) spectral power was formed in the left anterior temporal region.

**Conclusions:**

While the observed EEG slowdown in the left anterior temporal region is associated with clinical improvement, it is considered to be not pathological but reflects the adaptive neurophysiological processes associated with the restoration of emotional regulation, shift the brain activity into a mode more adequate for these patients and ensuring the suppression of depressive symptoms. Probably it is due to changes in the activation balance between the dorsolateral prefrontal and orbitofrontal cortical regions of the left hemisphere.

**Disclosure of Interest:**

None Declared

